# Plan Quality Assessment of Fractionated Stereotactic Radiotherapy Treatment Plans in Patients With Brain Metastases

**DOI:** 10.3389/fonc.2022.846609

**Published:** 2022-03-08

**Authors:** Mihály Simon, Judit Papp, Emese Csiki, Árpád Kovács

**Affiliations:** ^1^ Department of Oncoradiology, Faculty of Medicine, University of Debrecen, Debrecen, Hungary; ^2^ Doctoral School of Health Sciences, University of Pécs Faculty of Health Sciences, Pécs, Hungary

**Keywords:** FSRT = fractionated stereotactic radiotherapy, brain metastases, conformity index (CI), linac based, VMAT = volumetric modulated arc therapy

## Abstract

**Background and Purpose:**

The treatment options available in the management of brain metastases includes fractionated stereotactic radiotherapy (FSRT) and stereotactic radiosurgery (SRS) treatments. FSRT treatments have proved to be useful mainly in the treatment of larger volumes. This study aims to evaluate the FSRT treatment technique used in our department based on various plan quality indices.

**Methods and Materials:**

24 treatment plans of 23 patients were analyzed. Volumetric modulated arc therapy (VMAT) plans were generated in line with the department protocol. The following parameters were extracted: Radiation Therapy Oncology Group conformity index (RTOG CI), Paddick conformity index (Paddick CI), gradient index (GI), quality index (Q), homogeneity index (HI), and V24.4 volume as a parallel index of V12 used at SRS plan evaluation.

**Results:**

Plan conformity was acceptable, RTOG CI mean was 0.942; Paddick CI mean was 0.824. The mean GI value was 6.146. The mean of HI and Q indices were 1.263 and 0.94, respectively. V24.4 mean was 33.434 cm^3^. All plans achieved clinically acceptable organs-at-risk (OAR) constraints. PTV volumes were clustered into either 10 cm^3^ or 15 cm^3^ bins depending on the plan quality metric we used. The mean values show a balanced distribution of plan indices along the various PTV bins.

**Discussion:**

Our results based on the derived indices show that our FSRT approach can achieve clinically acceptable treatment plans. Furthermore, the clustering of PTV volumes show that these plan quality metrics remain acceptable for a wide spectrum of PTV volumes.

## Introduction

Brain metastases (BM) are considered to be a serious problem in the course of oncological diseases as 20-40% of patients affected by cancer will develop BMs (1). Although any primary site can metastasize to the brain, the most common sites are the lung (especially small cell lung cancer), breast, colon (colorectal cancer), skin, and kidney (2). Until recently, the major treatment option for these patients was whole brain radiotherapy (WBRT), but today the treatment for BMs depends on the number and size of lesions, on the patients performance state and the oncological status of the primary disease (3, 4). In cases with a limited number of metastases, stereotactic radiosurgery (SRS) was available, but due to the ablative nature of the treatment, the size of individual metastases was usually limited to 3.5 cm, and for multiple mets it was limited to 2.5 cm (5). SRS is a very high precision treatment given in one fraction in multiple static or moving beams to create a steep dose fall-off to spare normal brain tissue or adjacent organs at risk (OAR). High single doses near critical structures or large tumours are associated with significant risk of toxicity, and cognitive impairment has been associated with the brain volume receiving at least 12 Gy (6). There has been an attempt to utilize hypo fractionated stereotactic radiotherapy (FSRT) to achieve similar local control with acceptable toxicity rates. FSRT also uses high precision radiation therapy to treat BMs with non-invasive head fixation devices. The aim of FSRT is to minimize the normal tissue toxicity while preserving local control. It lacks the precision of SRS with the head frame, but can decrease the margins; therefore, in addition to decreasing normal tissue dose, it also makes use of the advantage of fractionation. Recent data suggest FSRT could be safer near critical structures and more effective than single fraction SRS (7, 8). In the present study we propose an FSRT treatment method implemented at our clinic. Plan quality indices are compared to values found in literature.

## Materials and Methods

### Patients

Between May 2019 and May 2020, 24 patients were treated using FSRT treatment at our department. Patients who had single or multiple intact BMs were selected for this study. Patients who underwent surgical resection, but also had intact lesions treated with FSRT were also included. All patients had a multidisciplinary board meeting indicating FSRT prior treatment. The number of lesions was between 1 and 7, and all of them were treated with a mono isocentre technique within the same treatment plan.

### Localization

Patients were immobilized in a supine position with individual open face thermoplastic head masks (QFix, Avondale, PA, USA),lying on a baseplate and a mouldable head cushion (QFix, Avondale, PA, USA). These masks are 2.4 mm thick and are strengthened with Kevlar to increase cranial support. Open face masks leave the eyes and nose free, and are proven to reduce anxiety, and increase patient comfort (9). The localization CT was performed on a Philips Brilliance Big Bore CT (Philips, The Netherlands) with a specific protocol with 2 mm slice thickness. Prior to CT, preferably within 2 weeks, all patients had an MRI scan of the brain with gadolinium contrast.

### Treatment Planning

All patients were contoured and planned on Pinnacle (Philips, The Netherlands) treatment planning system version 9.8. Before planning, a gadolinium contrast agent enhanced MRI was performed, and T1 post-contrast enhanced, and T2-weighted series were co-registered to the localization CT series with a rigid transformation. The GTV was contoured on the planning CT series based on MRI information; the department protocol defines GTV as the visible, contrast enhanced tumour volume on the T1 series. GTV was assumed to be equal to the clinical target volume (CTV). The GTV to PTV margin was an isotropic 3 mm. The OARs considered during planning were brainstem, optic chiasm, inner ears, lenses, optic nerves, eyes, and brain. Dose tolerances for the OARs are listed in **Table 1** (10–14). Single isocentre volumetric modulated arc therapy (VMAT) treatment plans were generated in the treatment planning system using 5 half arcs. The beam arrangement involved 2 coplanar and 3 non-coplanar half arcs (180 degrees); these required angular table movements (0°, ± 45°, +90°). In cases where the PTV or PTVs were localized laterally, one coplanar arc could be omitted to decrease overall brain dose.The isocentre was placed at the centre of mass of all the PTVs. A PRV margin of 3 mm was added to all serial organs at risk. The treatment energy was 6MV, and planners were allowed to use Flattening Filter Free (FFF) mode. The use of FFF mode in brain SRS has been described by several authors (15, 16). During treatment planning a PRV was generated with a 3 mm margin around each OAR (17).

**Table 1:** OAR tolerances for FSRT planning.

		Constraint
OAR		
	Brainstem	Dmax ≤ 20Gy
	Optic nerves	Dmax ≤ 15Gy
	Chiasm	Dmax ≤ 15Gy
	Lens	Dmax ≤ 8Gy
	Inner ears	Dmax ≤ 30Gy
	SpinalCord	Dmax ≤ 30Gy

### Prescription

The prescribed dose was 30 Gy, either in 5 or 6 fractions. Dose was prescribed to the isocentre (single lesion) or to PTV mean (multiple lesions). Initially the prescription was 80% of the isocentre dose or the PTV mean dose. Planners could shift the prescription between 70% and 95% to conform to the 95% isodose line to the PTV.

### Image Guidance and Treatment

Patients were treated and image guided on an Elekta Versa HD linear accelerator with HexaPod robotic treatment couch. A high resolution3D CBCT was acquired and registered to the localization CT series with an automatic bone match of a region, involving the entire skull. Before treatment, translational and rotational errors were corrected; thus, the residual error was significantly reduced. After the registration procedure a second 3D CBCT was performed to make sure that the position of the patient has not changed during the registration, and the patient is in the correct position for the treatment. The delivery started with the two coplanar beams, and the following arcs were scheduled in a manner that the gantry stop position of a half arc was the stat position of the following beam. This reduced the treatment time, and decreased the risk of positional deviations (18).

### HexaPOD

The Medical Intelligence HexaPODtm couch top (Schwabmünchen, Germany) is a specifically designed robotic treatment couch top rigidly mounted on the standard treatment table. It has 6 degrees of freedom, and can correct both translational and rotational errors. The range of linear movement of the table is +-30, +-30; +-40 mm in longitudinal, lateral, and vertical directions, respectively. Also, it has an angular range of +-3 degrees along all three rotational axes, which are denoted as pitch, roll and yaw. It has sub-millimetre and sub-degree-level accuracy, and is capable of efficiently moving the target (19, 20). Rotational errors of single isocentre plans treating multiple lesions were shown to increase the risk of compromised coverage above 0.5° (21).

### Analysis

The following indices were extracted and analyzed per patient across all patient plans: RTOG Conformity index (RTOG CI), Paddick Conformity Index (Paddick CI), Gradient Index (GI), Homogeneity Index (HI), and Quality Index (Q). The RTOG CI was defined as the ratio of the reference isodose volume (PIV) to the target volume (TV) (Eq 1.).


(Eq. 1.) $$CI_{RTOG}=\frac{PIV}{TV}$$

The Paddick CI is calculated by the ratio of the square of the volume of the target enclosed by reference isodose volume (TV_PIV_) to the multiplication of the target volume (TV) with the reference isodose volume (V_RI_) (Eq 2.).


(Eq. 2.) $$CI_{Paddick}=\frac{TV_{PIV}^{2}}{\left(TV*V_{RI}\right)}$$

The Gradient Index defined as the ratio of the 50% isodose (V50) volume to the prescription isodose volume (PIV) (Eq 3.).


(Eq. 3.) $$GI=\frac{V_{50}}{P_{IV}}$$

By definition, Homogeneity Index is the ratio of the maximum dose in the target volume (Imax) to the reference isodose volume (P_IV_) (Eq 4.)


(Eq. 4.) $$HI=\frac{I_{\max}}{P_{IV}}$$

The Q index is the ratio of the minimum dose given to the target volume (Imin) to the reference isodose volume (PIV) (Eq 5.).


(Eq. 5.) $$Q=\frac{I_{\min}}{P_{IV}}$$

Furthermore, the V24.4Gy parameter was extracted, V24.4 Gy is the volume of the brain which receives at least 24.4 Gy. V24.4 Gy was used to parallel the V12 index used in SRS techniques based on Eq 6.


(Eq. 6.) BED=nd(1+dαβ)

Here α/β = 2, n refers to the number of fractions, and d represents the fractional dose.

The PTV volumes were binned into 10cm^3^ bins with the corresponding indices.

## Results

**Table 2** summarizes patient characteristics. Age was in a range from 42 to 80, with a median age of 63.9 years. Patients were in a good overall condition:15 of them (63%) was ECOG 0, and only 2 patients (8%) were ECOG 2. The RPA classification of the disease ranged from 1 to 3. Altogether 4 patients (17%) received some form of intracranial irradiation before the SRT treatment, 1 patient had WBRT, 2 patients had SRS with a gamma knife, and 1 patient had received SRT previously at our institute for different lesions. These previous treatments were taken into account during the preparation and planning of the present treatment plans in terms of critical structure doses. 4 patients underwent surgical resection prior treatment; 7 patients had solitary lesions, and 17 patients had oligometastatic disease. The total number of lesions was 65. Most frequent primary sites were NSCLC (non-small cell lung cancer) (46%), breast (17%) and melanoma (13%) The mean number of treated lesions per patient was 2.7, with a standard deviation of 1.7.

**Table 2:** Patient characteristics.

Gender		
Male	13	54%
Female	11	46%
Age		
Min	42	
Max	80	
Median	63.9	
Range	37,4	
ECOG		
0	15	63%
1	7	29%
2	2	8%
RPA		
1	7	29%
2	12	50%
2a	4	17%
3	1	4%
Number of lesions		
1	7	32%
2	5	23%
3	3	14%
4	2	9%
5	3	14%
6	1	4%
7	1	4%
Primary site		
Breast	4	17%
Lung (NSCLC)	11	46%
Melanoma	3	13%
Other	4	17%
Previous intracranial radiotherapy		
No	20	83%
Yes	4	17%
Prior resection of brainlesion		
Yes	4	17%
No	20	83%

**Table 3** contains the laterality and localization specific lesion statistics. Out of 65 lesions, 32 were left sided and 32 were right sided, and laterality was not applicable for one lesion. In terms of localization, most of the metastases was supratentorial (45), 25% (18) was infratentorial, and 3% (2) were in the base of skull.

**Table 3:** Lesion charactheristics.

Laterality			
N/A		1	2%
Left		32	49%
Right		32	49%
		
Localization			
Supratentoral		44	68%
Infratentoral		19	29%
Base		2	3%

The conformity of the plans was calculated based on several indices, such as RTOG CI, Paddick CI, GI, HI, and Q. The RTOG CI, Paddick CI, and GI were extracted by patient (**Table 4**).

**Table 4:** RTOG conformity, paddick conformity, and gradient indices.

Different conformity index statistics across plans			
	RTOGCI	PaddickCI	GI
Mean	0.942	0.824	6.146
Std. Deviation	0.153	0.090	3.085
Range	0.631	0.299	14.448

Q and HI were calculated on each lesion (**Table 5**).

**Table 5:** Quality and homogeneity indices.

Quality and Homogeneity indices across lesions		
	Q	HI
Mean	0.940	1.263
Median	0.964	1.255
Std. Deviation	0.118	0.103
Range	0.757	0.496

The RTOG CI mean value was 0.942 with a standard deviation (SD) of ±0.153. The Paddick CI mean value was 0.824 with an SD of ±0.090. GI mean was 6.146±3.085 (SD) with a median of 5.483. The mean value of Q index was 0.940 with a 0.118 SD. HI mean was 1.263, the SD was 0.103.

**Figure 1:**
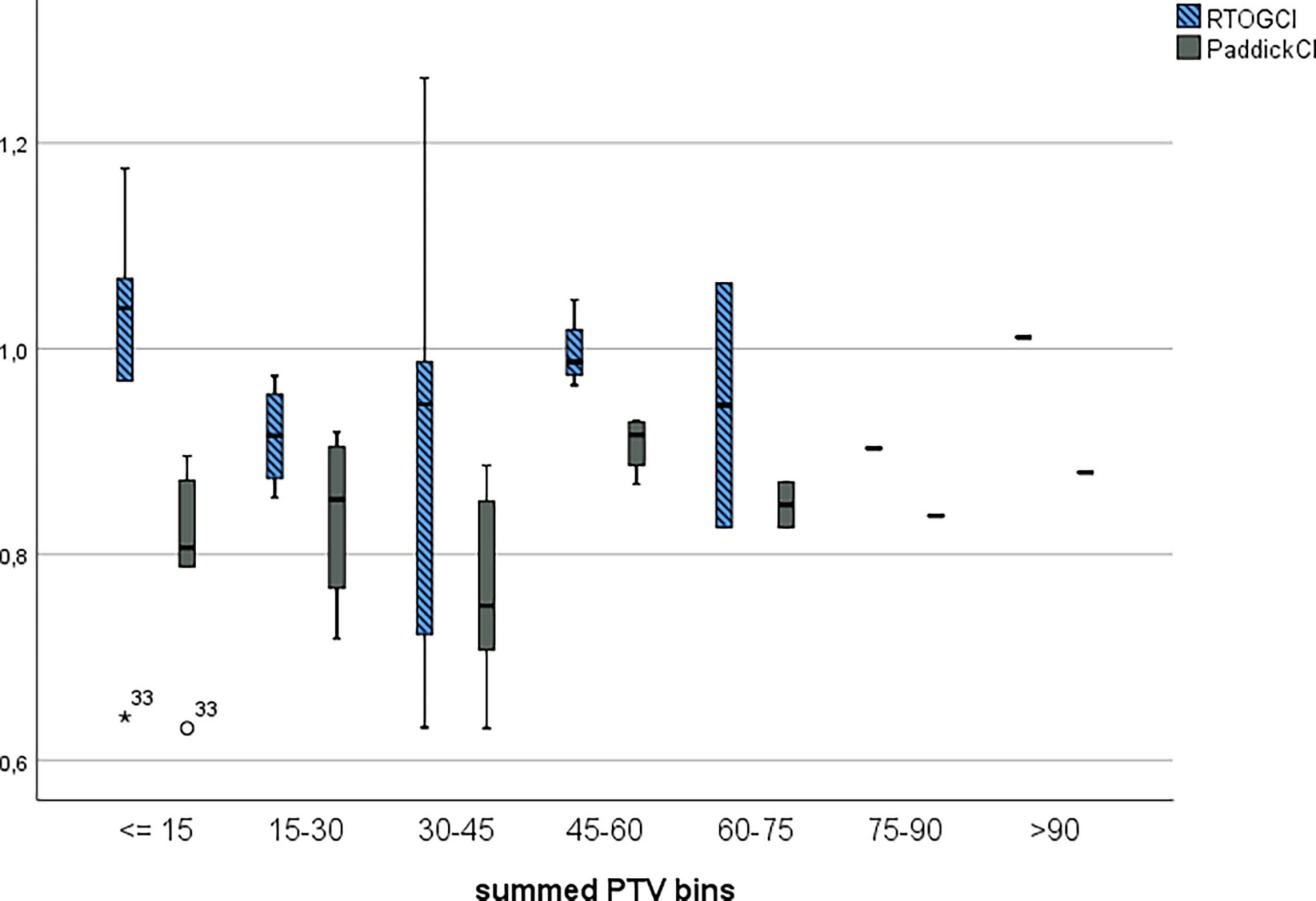
RTOG conformity and Paddick Conformity indices clustered into 15 cm^3^ bins.

**Figure 1** divides the RTOG CI and Paddick CI values per overall PTV volume for each patient. **Table 6** shows the conformity and gradient indices for each PTV cluster.

**Table 6:** RTOG, paddick conformity, and gradient indices for PTV clusters.

RTOG, Paddick, and Gradient indices for PTV bins							
	<= 15	15-30	30-45	45-60	60-75	75-90	>90
RTOGCI	0.979	0.915	0.894	0.996	0.9450	0.903	1.011
PaddickCI	0.799	0.836	0.769	0.908	0.8483	0.838	0.880
GI	9.185	6.959	5.334	4.495	3.7289	6.586	4.379

**Figure 2:**
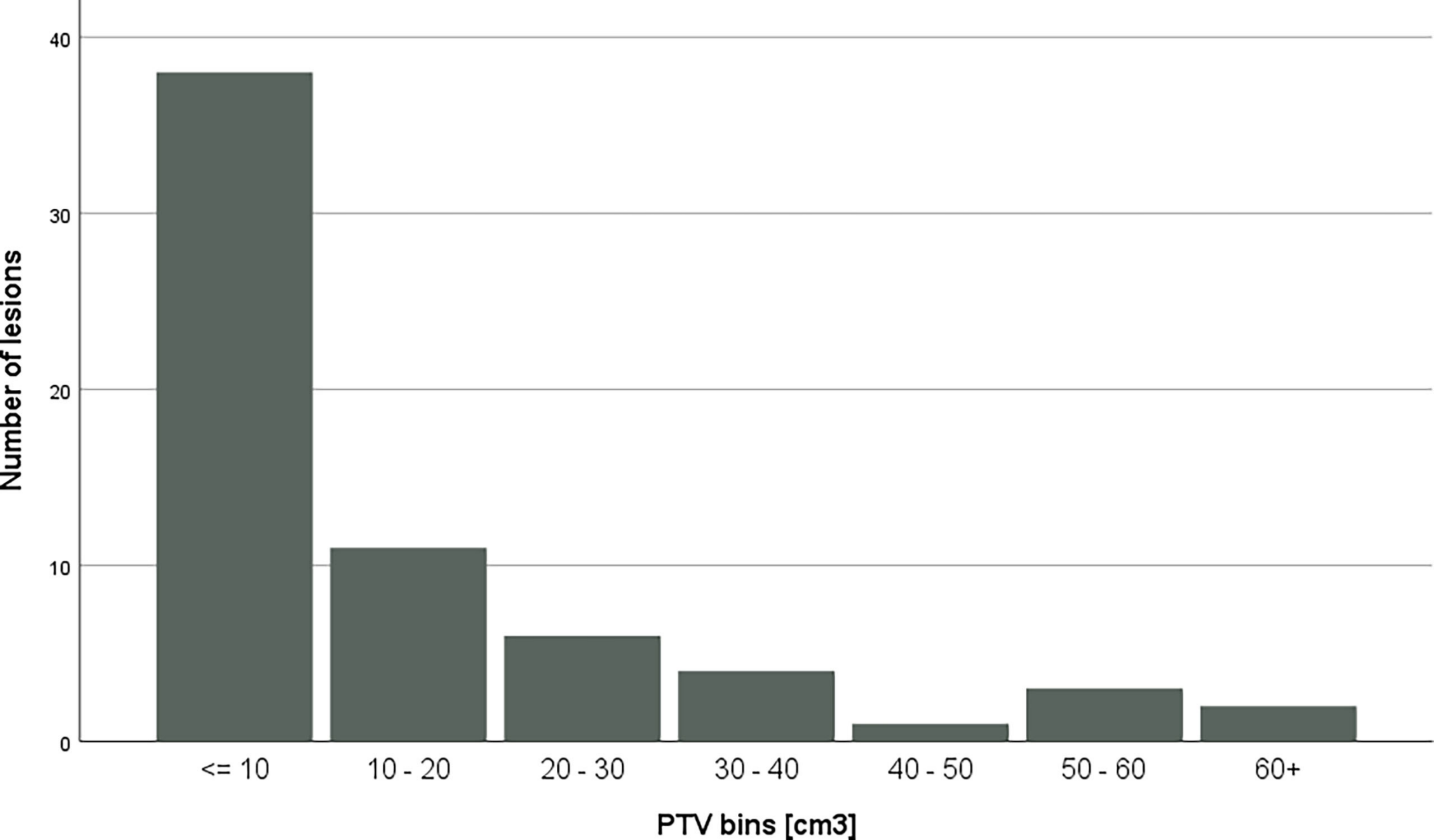
Distribution of PTV volumes across bins. Each PTV had been assigned to a bin with according to the volume.

**Figure 3:**
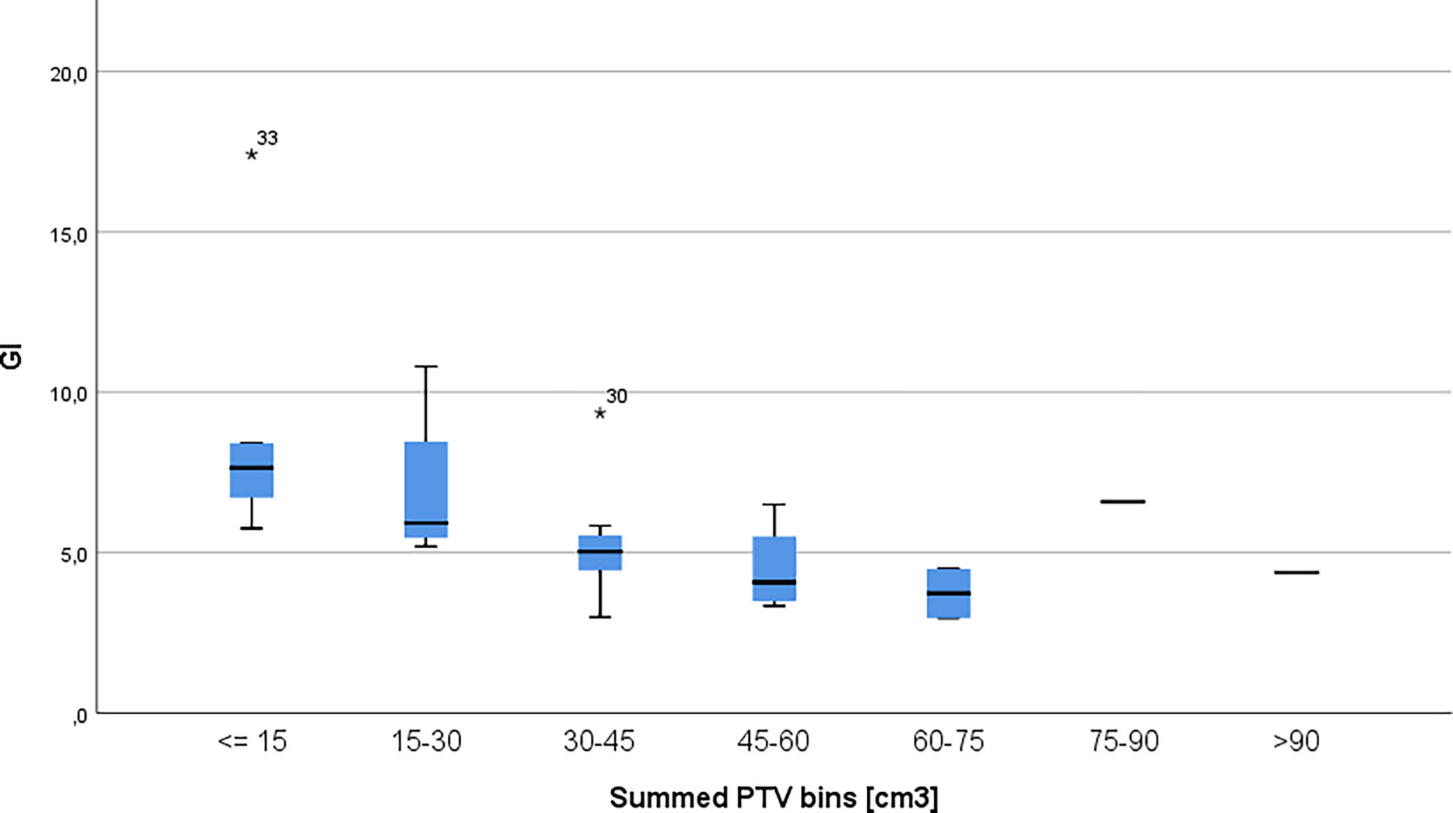
Gradient index per patients clustered in 15cm^3^ bins. The * symbol is the most extreme value compared to the mean for that dataset.

PTV volumes ranged from 1.23 cm^3^ to 61.63 cm^3^; the mean volume was 14.17 cm3 with a standard deviation of 16.5 cm^3^, while the median of the volumes was 4.89. **Figure 2** shows the distribution of PTV volumes across all metastases. **Figure 3** shows the GI values clustered by the overall PTV volume per patient.

**Figure 4:**
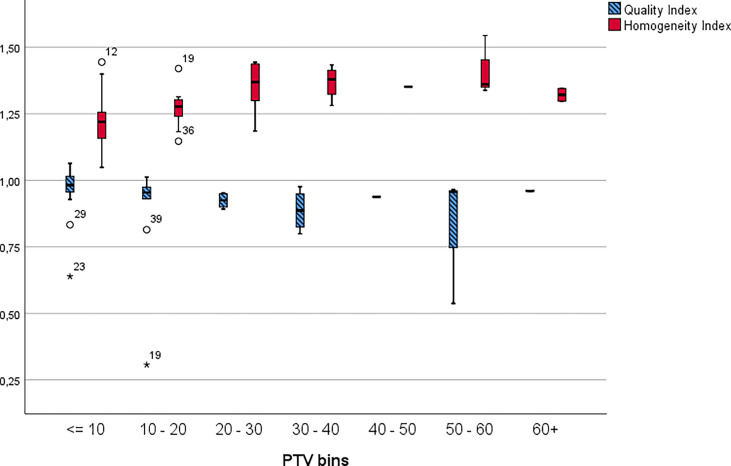
Homogeneity and quality indices by lesion clustered into 10cm^3^ bins. The * symbol is the most extreme value compared to the mean for that dataset.

**Figure 4** displays the HI and Q clustered by lesion size. **Table 7** presents the Q and HI values for the individual PTV volume bins.

**Table 7:** Quality and homogeneity indices for individual PTV volume bins.

Quality and homogeneity indices for PTV bins							
	<= 10	10 - 20	20 - 30	30 - 40	40 - 50	50 - 60	60+
Q	0.974	0.883	0.924	0.887	0.9380	0.820	0.960
HI	1.215	1.272	1.351	1.368	1.3513	1.414	1.321

Our results show that the applied treatment planning technique was able to generate clinically acceptable plans in terms of these metrics across various size of metastases.

V24.4 was calculated after the exclusion of the given PTV volume from the 24.4 Gy area; for each lesion a 24.4 Gy volume was defined by dividing the 24.4 Gy area of the patient by the number of treated metastases. **Table 8** contains the values for V24.4Gy.

**Table 8:** V24.4Gy descriptive statictics.

V24.4 Gy values for plans	V24.4
Minimum	9.233
Maximum	77.230
Mean	33.434
Std. Deviation	17.688

## Discussion

Stereotactic radiosurgery (SRS) is the gold standard treatment of brain metastases (BM); however, there is a limitation in treatable tumour size (<4 cm) and the risk of radio necrosis increasing with doses over 12Gy (11). Some comparative analyses have been published on SRS and FSRT (22–24), suggesting FSRT may offer an alternative solution due to its comparable precision during treatments, as well as its ability to treat larger lesions. Regarding tumour progression and risk of radio necrosis, Putz et al. published better results for FSRT that single fraction SRS (25). The overall findings of our study have demonstrated the plausibility of using our FSRT treatment technique in the treatment of multiple brain metastases. We proposed a linac-based FSRT treatment method with non-invasive patient positioning and treatment planning, which can achieve clinically acceptable conformality based on various indices. Linac-based techniques proved to have improved conformity over gamma knife (GK) treatments in large lesions, and even compete GK in terms of V12 volume (26). Our aim was to derive conformity indices used in stereotactic treatments such as RTOG conformity index (RTOG CI), Paddick conformity index (Paddick CI), Quality index (Q), Homogeneity index (HI) (27),and biologically equate the V12 volume used in SRS technique based on Equation 6. Milano et al. paralleled the V12 index used in SRS techniques with V24.4 for 5 fraction FSRT treatments (11). Hsu et al. compared different treatment modalities based on similar indices based on tumour size and tumor distance from brainstem. Our mean PTV volume was 14.17cm^3^, the corresponding values for FFF-VMAT plans were 0.73 and 0.72 for Paddick CI, and 1.19 and 1.17 for HI. Our mean Paddick CI and HI values were 0.824 and 1.263, respectively. Ruggieri et al. evaluated treatment plans generated with HyperArc™ (HA) (Varian Medical System, Palo Alto, CA) and MultipleBrainMets™ (MBM) (Brainlab AG, Munchen, Germany) in terms of plan conformity. The mean number of treated lesions was 5. The mean Paddick CI values for HA and MBM plan were 0.94 and 0.75, respectively (28, 29). For RapidArc (RA) plans Ruggieri reported 0.87 and 6.08 for Paddick CI and GI values, respectively. Our gradient index (GI) mean value was 6.146. Individual PTV volumes were clustered into 10cm^3^ bins, and the derived mean of Q and HI indices shows good consistency among the various sized bins. In terms of RTOG CI, Paddick CI, and GI the overall PTV volumes were binned into 15cm^3^ bins. The results show consistent values of these indicators as well, regardless of PTV volume. In terms of V12 Ruggieri et al. published 23.7cm^3^ and 37.3cm^3^ for HA and MBM plans, and 42,2cm^3^ for RA plans, respectively. In our cohort the mean value for V24.4Gy was 33.434 cm^3^.

The main limitation of this study is the limited number of patients and the lack of comparability to other treatment techniques such as GK.

In summary, our approach to treat multiple brain metastases with a single isocenter VMAT technique using non-invasive mask fixation proved to be clinically plausible and based on the metrics the results are comparable to the results published in literature. Furthermore, our approach can reach clinically acceptable values for plan quality indices in a wide spectrum of PTV volumes.

## Data Availability Statement

The raw data supporting the conclusions of this article will be made available by the authors, without undue reservation.

## Author Contributions

MS: data collection, statistical analyses, manuscript preparation. JP and EC: tables, data collection, manuscript preparation. ÁK: manuscript preparation. All authors contributed to the article and approved the submitted version.

## Conflict of Interest

The authors declare that the research was conducted in the absence of any commercial or financial relationships that could be construed as a potential conflict of interest.

## Publisher’s Note

All claims expressed in this article are solely those of the authors and do not necessarily represent those of their affiliated organizations, or those of the publisher, the editors and the reviewers. Any product that may be evaluated in this article, or claim that may be made by its manufacturer, is not guaranteed or endorsed by the publisher.
